# PHQ-9 and PHQ-2 for Screening Depression in Chinese Rural Elderly

**DOI:** 10.1371/journal.pone.0151042

**Published:** 2016-03-15

**Authors:** Zi-wei Liu, Yu Yu, Mi Hu, Hui-ming Liu, Liang Zhou, Shui-yuan Xiao

**Affiliations:** Department of Social Medicine and Health Management, Xiangya School of Public Health, Central South University, Changsha, Hunan, China; Federal University of Rio de Janeiro, BRAZIL

## Abstract

**Objectives:**

This study aimed to explore cut-off scores of the 9-item Patient Health Questionnaire (PHQ-9) and 2-item Patient Health Questionnaire (PHQ-2) for depression screening in Chinese rural elderly.

**Methods:**

A cross-sectional study was conducted on 839 residents aged 60 years and above in rural areas of Liuyang County. PHQ-9 was adopted to evaluate depression. The Structured Clinical Interview for DSM Disorders (SCID-I) was adopted to diagnose major depressive disorder (MDD) as a golden standard. Sensitivity, specificity, positive and negative predictive value, positive and negative likelihood ratio, Youden’s index and the receiver operating characteristic (ROC) curve were analyzed on PHQ-9 and PHQ-2.

**Results:**

The Cronbach's alphas of PHQ-9 and PHQ-2 were 0.82 and 0.76, respectively. The score of 8 of the PHQ-9 showed the highest Youden’s index of 0.85, with a sensitivity of 0.97 and specificity of 0.89 respectively, and the area under the ROC curve (AUC) was 0.97 (95% CI: 0.96–0.98). The score of 3 of PHQ-2 showed the highest Youden’s index of 0.79, with both sensitivity and specificity were 0.90 and the AUC was 0.94 (95% CI: 0.90–0.97).

**Conclusions:**

Both PHQ-9 and PHQ-2 are valid screening instruments for depression in the rural elderly in China, with recommended cut-off scores of 8 and 3 respectively.

## Introduction

Depression is common among the elderly. In China, the prevalence of depressive symptoms was 22.7% among the elderly, rural areas higher than urban areas [1]. Depression is associated with various severe health-related outcomes as function impairment and suicide [2–5]. Catastrophic outcomes are preventable if depression was detected and treated timely and appropriately. Because of too complicated and time consuming, questionnaires as Geriatric Depression Scale (GDS), Beck Depression Inventory (BDI), Centre for Epidemiologic Studies Depression Scale (CES-D), hindered the effect and efficiency of depression screening among rural elderly [6–8]. PHQ-9 is relatively favored on depression screening among rural elderly due to its simplicity and time efficiency [9, 10].

The PHQ-9 is a brief, self-explanatory questionnaire developed for depression symptoms evaluation [11]. The items were designed according to the diagnosis criteria of major depressive disorder (MDD) in the Diagnostic and Statistical Manual of Mental Disorders, 4th edition (DSM-IV) [11]. PHQ-9 had been used widely in rural elderly due to its good reliability and validity in this population [12–15]. PHQ-2, the abridged version of PHQ-9, was composed of the first two items of the PHQ-9 [16]. There is evidence showing good reliability and validity of PHQ-2 in urban population, but none in rural elderly [17].

A cut-off score of PHQ-9 is used to differentiate between a subject with or without MDD for the purpose of early intervention. Determining a cut-off score should not only consider indicators such as sensitivity and specificity, but also the aims and settings of utilization. Ten points as the cut-off score for depression screening was usually adopted in most studies in China [12, 18–20]. One study conducted in the general population in Shanghai recommended 7 points as the cutoff of PHQ-9 [21]. Another study conducted in urban elderly of Hangzhou suggested the cut-off score of PHQ-9 at 9 [17]. For PHQ-2, the score of 3 was recommended as the cutoff for screening depression in most published studies [12, 20, 22, 23]. However, there is little evidence to recommend optimal cut-off values of PHQ-2 and PHQ-9 in rural areas of China. Considering the differences in social-economic situation and depression-related factors between rural and urban elderly [13, 24], the aim of the present study was to fill in the knowledge gap by exploring cut-off scores of PHQ-9 and PHQ-2 for detecting depression among rural elderly in China.

## Materials and Methods

### Study setting

Liuyang County locates in the northeast of Hunan Province, China, with a population of 142 million. The number of people aged 60 and above was 194,000 in the year of 2010 [25]. Administratively, Liuyang is divided into 4 districts in urban areas and 33 towns in rural areas. The average annual family income in Liuyang was approximately 24,236 CNY ($3,740 USD) in urban areas and 13,193 CNY ($2,036 USD) in rural areas in 2011 [26].

### Design and participants

**Fig 1:**
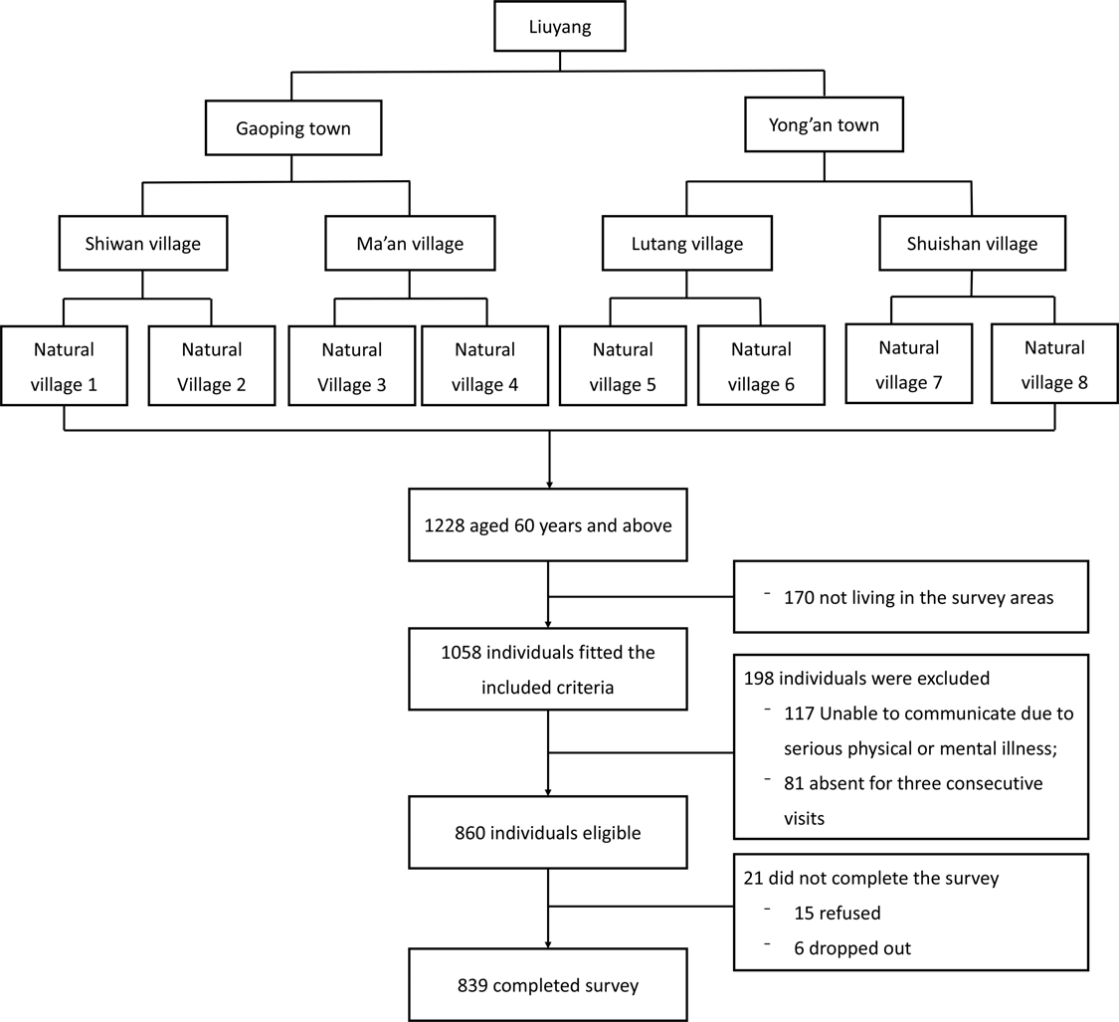
Flow chart of participant’s enrolment.

This was a cross-sectional study, conducted as a part of “the 2010 National Science and Technology Support Program: The Assessment, Warning and Intervention Study on the Emotional Problems of the Chinese Population”. Ethics approval was granted by the Institutional Review Board of the Xiangya School of Public Health, Central South University. The target population was residents aged 60 and above who have lived in rural areas of Liuyang for over 6 months. Eligibility criteria of participants included being 60 years of age and above at the time of interview and a resident in the survey site for half a year or more. A multistage cluster-sampling method was adopted to identify subjects (Fig 1). In the first stage, two towns (Gaoping and Yong’an) were randomly selected from 33 towns in the rural areas. In the second stage, two administrative villages were randomly selected from each town. Administrative village was the basic administrative organization in the rural areas, composed by several geographically adjacent natural villages. In the third stage, two natural villages were randomly selected from each administrative village. Natural villages refer to villages that were naturally formed by residents living together for a long time in a certain natural environment. Finally, all elderly (n = 1228) within eight natural villages were invited to take part in the study. Those who were not living in the areas during the research period, those with difficulty in communication due to serious physical or mental illness were excluded, resulting in a final sample of 860 residents. Among the 860 subjects, 15 refused to participate and 6 quit the study midway. In sum, 839 subjects completed the surveys with a response rate of 97.6%.

### Procedure

The survey was conducted from November 2010 to August 2011. Interviewers were composed of eight postgraduate students with medical education background, and two psychiatrists with SCID training experience. One of the psychiatrists qualified as SCID trainer was responsible for SCID training of all interviewers. All investigators had received consistent training before the investigation. Investigator training included understanding the objectives of the study, scales, the principle and requirements of interview, skills of asking questions and use of words. Interviewers conducted face-to-face interviews with each participant in their household after obtaining written informed consent. Approximately one hour was spent for the total interview and each household was reimbursed with small gifts such as kitchen utensils (equivalent to about USD $2). Since the first two items of the PHQ-9 were extracted to comprise the PHQ-2, we only administered the PHQ-9 and did not administer PHQ-2 separately. After the respondents completed the survey, a quality control person checked all information from interviews to ensure that there were no inconsistencies or missing items.

### Instruments

#### PHQ-9

The PHQ-9 is a nine-item scale which was used to assess depressive symptoms. Each item in PHQ-9 asked about the frequency of a depressive symptom experienced in the two weeks prior to survey administration. The score of each item ranges from 0 (never) to 3 (almost every day) and the total score is 27. We used the Chinese print version of PHQ-9 in this study [27].

#### SCID-I

The SCID-I is a semi-structured diagnostic interview that is used to determine DSM-IV Axis I disorders (major mental disorders). The major depressive disorder episode was used for diagnosis of MDD. The Chinese version of SCID-I has been validated in the Chinese population [28, 29]. In this study, the result obtained through MDD module in SCID-I was considered as a gold standard. The current version of the SCID-I is based on the DSM-IV. The diagnostic criteria closely resemble those of the DSM-V.

### Statistical analysis

Statistical analyses were performed using SPSS 13.0 software (SPSS/IBM, Chicago, IL). Cronbach’s alpha coefficients were calculated for assessing the internal consistency of PHQ-9 and PHQ-2. The distribution of data was identified by histogram plot combined with Kolmogorov-Smirnov test. The total score of PHQ-9 and individual item scores were treated as a continuous variable and ordinal variables respectively. The relationship between the total score and individual items was explored by Speaman’s correlation analyses. We used receiver operating characteristic (ROC) curve analysis to measure the overall accuracy of the tools, and used sensitivity, specificity, positive predictive value (PPV), negative predictive value (NPV), positive likelihood ratio (PLR), and negative likelihood ratio (NLR) to evaluate the validity of the tools. Cutoffs scores balancing sensitivity and specificity were determined by using the Youden index, which was calculated by (sensitivity + specificity– 1) [30, 31].

## Results

### Participant characteristics and scores on the PHQ-9

The distribution of socio-demographic characteristics of the 839 subjects is shown in Table 1. Subjects ranged in age from 60 to 90 years with a mean age of 69.0 ± 7.1 years (±SD). Four hundred and forty eight (53.4%) were male and 391 (46.6%) were female. Diagnosed by the SCID-I, the prevalence of MDD was 6.8% (CI: 5.1% − 8.5%). The total score of PHQ-9 ranged from 0 to 27 with a median score of 2. The median score of each item was 0, with inter-quartile range of 0–1. The total score of PHQ-2 ranged from 0 to 6 with a median score of 0.

**Table 1:** Features of subjects (*N* = 839).

Variables	Non-depression% (n)	Depression ^a^% (n)
**N**	93.2 (782)	6.8 (57)
**Age** ^b^	68.9±7.1	70.7±7.2
**Gender**		
Male	53.1 (415)	57.9 (33)
Female	46.9 (367)	42.1 (24)
**Educational level**		
Below primary school	26.1 (204)	33.3 (19)
Primary school	61.3 (479)	61.4 (35)
Middle school	12.6 (99)	5.3 (3)
**Marital status**		
Married (including remarried)	73.1 (572)	59.6 (34)
Never married	2.2 (17)	3.5 (2)
Divorce	1.3 (10)	1.8 (1)
Windowed	23.4 (183)	35.1 (20)
**Physical diseases** ^c^		
Yes	56.0 (438)	87.7 (50)
No	44.0 (344)	12.3 (7)
**Self-reported income** ^d^		
Low	21.5 (168)	33.3 (19)*
Middle	54.7 (428)	63.2 (36)
High	23.8 (186)	3.5 (2)

^a^ Diagnosis from SCID-I

^b^ Unit is years.

^c^ The information of physical disease was collected from self-report of subjects.

^d^ Self-reported personal annual average income was classified based on the 2009 national poverty line (≤1196 Yuan per year) for a low income level and the 2010 farmers’ per capita income in Hunan Province (≥5523 Yuan per year) for a high income level.

* *P* = 0.001.

### Reliability and item analysis

The PHQ-9 had a Cronbach’s alpha of 0.82, with the correlations between the total score and each item ranging from 0.45 to 0.71(p < 0.001) (see Table 2). Cronbach’s alpha of PHQ-2 was 0.76. The correlations between the total scores of the PHQ-2 and each item were 0.81 and 0.90 (p < 0.001), respectively.

**Table 2:** Item analysis of PHQ-9.

PHQ-9 items	Item-total correlation	Cronbach's alphaif item is deleted
(1) Little interest or pleasure in doing things	0.61	0.80
(2) Feeling down, depressed, or hopeless	0.72	0.78
(3) Trouble falling or staying asleep, or sleeping too much	0.69	0.81
(4) Feeling tired or having little energy	0.68	0.80
(5) Poor appetite or overeating	0.56	0.81
(6) Feeling bad about yourself—or that you are a failure	0.52	0.81
(7) Trouble concentrating on things	0.45	0.82
(8) Moving or speaking so slowly that other people could have noticed	0.47	0.81
(9) Thoughts that you would be better off dead or of hurting yourself	0.45	0.81

### Cut-off score for PHQ-9

**Fig 2:**
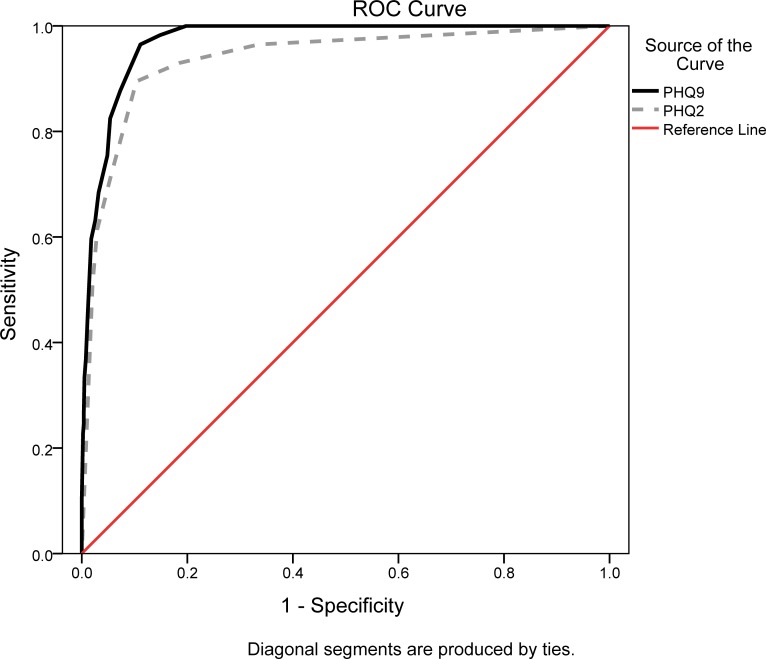
The receiver operating characteristic (ROC) curve of the PHQ-9 and PHQ-2 versus the SCID-I for a depression diagnosis.

For PHQ-9, the sensitivity, specificity, PPV, NPV and likelihood ratios of different cut-off scores are presented in Table 3. The score of 8 on PHQ-9 showed the highest Youden’s index of 0.85, with sensitivity and specificity were 0.97 and 0.89 respectively. The area under the ROC curve (AUC) was 0.97 (standard errors = 0.01, 95% confidence interval: 0.96–0.98), which also supported the criterion validity of PHQ-9 at the score of 8 (Fig 2).

**Table 3:** Sensitivity, specificity, predictive values, and likelihood ratios at various cut-off scores of the PHQ-9.

PHQ-9 score	Sensitivity	Specificity	PPV	NPV	PLR	NLR
≥5	1.00	0.76	0.23	1.00	4.17	0.00
≥6	1.00	0.80	0.27	1.00	5.00	0.00
≥7	0.98	0.85	0.33	1.00	6.53	0.02
≥8	0.97	0.89	0.39	1.00	8.82	0.03
≥9	0.93	0.90	0.41	0.99	9.30	0.08
≥10	0.83	0.95	0.53	0.99	16.60	0.18
≥11	0.83	0.95	0.53	0.99	16.60	0.18
≥12	0.75	0.95	0.53	0.98	15.00	0.26
≥13	0.68	0.97	0.61	0.98	22.67	0.33
≥14	0.63	0.97	0.64	0.97	21.00	0.38
≥15	0.60	0.98	0.71	0.97	30.00	0.41

PPV: positive predictive value; NPV: negative predictive value; PLR: positive likelihood ratio; NLR: negative likelihood value.

### Cut-off score for PHQ-2

For PHQ-2, the sensitivity, specificity, and likelihood ratios of different cut-off scores are presented in Table 4. The score of 3 on PHQ-2 showed the highest Youden’s index of 0.79, with both sensitivity and specificity were 0.90. ROC curve analysis showed that AUC = 0.94, standard errors = 0.02, 95% CI: 0.90–0.97.

**Table 4:** Sensitivity, specificity, predictive values, and likelihood ratios at various cut-off scores of the PHQ-2.

PHQ-2 score	Sensitivity	Specificity	PPV	NPV	PLR	NLR
≥1	0.97	0.67	0.17	1.00	2.94	0.04
≥2	0.93	0.82	0.27	0.99	5.17	0.09
≥3	0.90	0.90	0.39	0.99	9.00	0.11
≥4	0.72	0.94	0.48	0.98	12.00	0.30

### Comparison of screening performance between PHQ-9 and PHQ-2

The Cronbach’s alpha was higher in PHQ-9 than in PHQ-2. The AUC was higher in PHQ-9 than in PHQ-2. PHQ-9 with 8 as cutoff showed higher sensitivity and accuracy than PHQ-2 with 3, but similar PPV, NPV, and PLR values as PHQ-2 with 3. When the score of 8 for PHQ-9 and 3 for PHQ-2 were adopted, 16.9% and 15.7% of subjects were detected to have possible depression.

## Discussion

In the present study, the results of this study suggest that the PHQ-9 and PHQ-2 are valid instruments for depression screening among the elderly population in the rural areas of China. Based on examination of indicators as Youden’s index, sensitivity, specificity and AUC, the score of 8 for PHQ-9 and the score of 3 for PHQ-2 were recommended as cut-off scores.

It has been proved that PHQ-9 was suitable for the elderly population with good reliability and validity [7, 9]. The score of 10 was commonly adopted as a cut-off score to distinguish individual with MDD from those without it in most cases [32]. In this study, the score of 8 showed the highest Youden’s index with a better balance between sensitivity and specificity than on the score of 10. This indicates PHQ-9 may have a better performance on identifying MDD among elderly in rural China when the cut-off set at 8. However, determining a cut-off score should not only consider indicators such as sensitivity and specificity, but also consider the aims and settings of utilization. When used as a screening instrument to identify elderly at high risk for MDD, there are potential dangers if subjects with MDD risk had not been identified [3, 5, 33]. Higher false positive rate was acceptable under this circumstance. According to our results, in this case scores of 6 and 7 could be adopted as cut-off, as the sensitivity of screening reached 1.00 and 0.98 respectively. On the other hand, when used in research purpose and high specificity is demanded, the scores of 8 or higher could be used as cut-offs to avoid too many false positives.

In our study, PHQ-2 had balanced sensitivity of 0.90 and specificity of 0.90 at the cut-off score of 3, which were consistent with previous studies in urban Chinese elderly [17] and in other population [20, 27, 34]. Based on results of the present study and previous evidence, the score of 3 is recommended for PHQ-2 to screen depression in rural elderly in China.

The screening performance of both PHQ-9 and PHQ-2 is good in identifying elderly with depression in rural areas. The choice of PHQ-9 or PHQ-2 should depend on the purposes and settings. As PHQ-2 only use the first two items of PHQ-9, the administrative time of it is markedly shorter than PHQ-9. When time is the priority of consideration, for instance used by busy primary care providers, PHQ-2 may be more suitable. In addition, when PHQ-9 on the score of 8 and PHQ-2 on the score of 3, the Youden’s index both reach to the best, which means they both have the highest performance on screening. However, all indicators, including sensitivity, Youden’s index and AUC higher in PHQ-9 than PHQ-2 indicating that PHQ-9 has a better accuracy than PHQ-2 and more suitable for research purposes.

There may be a limitation in this study. For each individual case, both PHQ-9 and SCID-I were conducted by the same interviewer. Results of SCID-I may be influenced by the PHQ-9 due to the priming effect. Although in our study PHQ-9 scores were not calculated during the interview, and there were 30 minute interval between the administration of PHQ-9 and the interview of SCID-I, priming effects could not be excluded completely.

## Conclusions

The results of this study suggest that the PHQ-9 and PHQ-2 are valid screening tools for depression in Chinese rural elderly, with a recommended cut-off score of 8 for the PHQ-9 and a cut-off score of 3 for the PHQ-2.
